# Airspeed-Aided State Estimation Algorithm of Small Fixed-Wing UAVs in GNSS-Denied Environments

**DOI:** 10.3390/s22093156

**Published:** 2022-04-20

**Authors:** Xiaoyu Ye, Yifan Zeng, Qinghua Zeng, Yijun Zou

**Affiliations:** 1 School of Aeronautics and Astronautics, Sun Yat-sen University, Shenzhen 518107, China; yexy39@mail2.sysu.edu.cn (X.Y.); zouyj5@mail2.sysu.edu.cn (Y.Z.); 2 School of Systems Science and Engineering, Sun Yat-sen University, Guangzhou 510275, China; zengyf29@mail2.sysu.edu.cn

**Keywords:** GNSS-denied navigation, nonlinear complementary filter, sensor fusion, the fix-wing UAV, nongravitational acceleration estimation, airspeed

## Abstract

Aimed at improving the navigation accuracy of the fixed-wing UAVs in GNSS-denied environments, this paper proposes an algorithm of nongravitational acceleration estimation based on airspeed and IMU sensors, which use a differential tracker (TD) model to further supplement the effect of linear acceleration for UAVs under dynamic flight. We further establish the mapping relationship between vehicle nongravitational acceleration and the vehicle attitude misalignment angle and transform it into the attitude angle rate deviation through the nonlinear complementary filtering model for real-time compensation. It can improve attitude estimation precision significantly for vehicles in dynamic conditions. Furthermore, a lightweight complementary filter is used to improve the accuracy of vehicle velocity estimation based on airspeed, and a barometer is fused on the height channel to achieve the accurate tracking of height and the lift rate. The algorithm is actually deployed on low-cost fixed-wing UAVs and is compared with ACF, EKF, and NCF by using real flight data. The position error within 30 s (about 600 m flying) in the horizontal channel flight is less than 30 m, the error within 90 s (about 1800 m flying) is less than 50 m, and the average error of the height channel is 0.5 m. The simulation and experimental tests show that this algorithm can provide UAVs with good attitude, speed, and position calculation accuracy under UAV maneuvering environments.

## 1. Introduction

Small fixed-wing unmanned aerial vehicles (UAVs) play an essential role in future air battles and civilian fields due to their low cost, small size, full autonomy, and dense formation. The normal autonomous flight of UAVs is inseparable from the accurate ground velocity and absolute position provided by the Global Navigation Satellite System (GNSS), and especially the real-time kinematic (RTK) measurement can provide centimeter-level position using the technique of dispersion assignment. The traditional navigation mode of the Inertial Navigation System (INS) assisted by GNSS is greatly challenged, since the GNSS is easily interfered with by complex external environments or human factors [1,2].

State estimation of UAVs can be defined as the process of tracking the current attitude, velocity, and position of the vehicle [3]. The design of the motion estimation algorithm is necessary for flight control and is a crucial step in the development of autonomous flying machines. The accuracy of the attitude and heading reference systems (AHRS) have a crucial role. With the continuous improvement in the chip manufacturing process, the current low-cost UAV airborne sensors mainly use microelectromechanical systems (MEMS), which are limited by the performance of low-cost sensors and inevitably introduce interference noise and random noise. The rapid development of modern wireless communication systems provides technical support for various UAV positioning systems [4], and provides good and fast data communication links for various positioning methods such as the Ultra-Wide Band (UWB) and GNSS, which rely on antenna to receive data [5]. The traditional GNSS/INS-integrated navigation of UAVs use GNSS positioning measurement information to estimate the bias of the gyroscope, accelerometer, and attitude error, then feedback [6]. According to the different fusion levels of the GNSS/INS-integrated navigation system, it can be divided into loose integration, tight integration, and deep integration [7]. This method can improve the accuracy of attitude estimation significantly. However, in GNSS-denied environments, the UAV cannot rely on GNSS positioning information for guidance and control, which has a great impact on the autonomous flight of the UAV.

In recent years, many researchers have been exploring autonomous navigation techniques in GNSS-denied environments to improve positioning and attitude accuracy. Visual-based position does not depend on external equipment support and has high autonomy. Visual and inertial navigation integration technologies have made great strides in recent years. Mourikis et al. [8] presented an EKF-based algorithm for real-time vision-aided inertial navigation. Tong Qin et al. [9] proposed a tightly coupled, nonlinear optimization-based method used to obtain highly accurate visual–inertial odometry by fusing preintegrated IMU measurements and feature observations, which successfully solved the navigation problem of rotorcraft in GNSS-denied environments. At present, fixed-wing UAVs have the following difficulties in navigating GNSS-denied environments: (1) High flying speed [10]: Higher speeds bring higher flight dynamics and vibrations caused by airflow, which puts higher requirements on the UAV damping design. The G-sensitivity of the gyroscope is more difficult to process directly at the algorithm level, and direct inertial integration will cause rapid dispersion of attitude position. (2) The high speed and high altitude of fixed-wing UAVs results in a vast field of view for the airborne camera. Consequently, capturing high-quality feature points becomes more complex, making it hard to apply vision-based inertial navigation algorithms to fixed-wing UAVs directly.

Most commonly used attitude estimation algorithms can be concluded to three kinds: the extended Kalman filter, the gradient descent algorithm, and the complementary filter algorithms. The EKF is more precise in the process of the state error transfer and the bias error, and the process noise of the gyroscope and accelerometer are modeled, thence the error parameters are estimated and compensated by other sensors, but it obviously adds computational complexity [11]. Leutenegger, S et al. [12] used an extended Kalman filter estimation framework to replace GPS updates with airspeed measurement under GPS-denied environments. The experiment demonstrates that the position error enlarges linearly with time. Despite its widespread use in UAV navigation, the EKF is subject to limitations. The local linearization of the process dynamic models and measurement models for feature points can degrade with the increasing nonlinearity in the system dynamics [13]. The gradient descent algorithm uses a quaternion representation, allowing the accelerometer and magnetometer data to be used in an analytically-derived and optimized gradient descent algorithm to compute the direction of the gyroscope measurement error as a quaternion derivative [14]. Based on the principle of dual vector gravity and magnetic field fixation in the complementary filters algorithm, the accelerometer data is considered as an approximate observation of the local gravity vector, and the accumulated error of the heading angle is corrected by magnetic field measurements [15,16]. Mahony, R. et al. [15] first proposed and established the complementary filter on the special orthogonal group (SO3) and proved the Lyapunov stability to ensure the global stability of the observer error. However, this algorithm is sensitive to nongravitational acceleration, which may lead to the wrong attitude correction in maneuvering environments. In [17,18], the GPS velocity measurement was used to establish the model of the nongravitational acceleration of the vehicle, and the covariance of the measured noise was increased in the absence of the GPS signal, which did not solve the problem of the accurate estimation of nongravitational acceleration under GPS-denied conditions. Euston, M. et al. [19] used airspeed measurement in vehicle nongravitational acceleration observation for the first time. By establishing the centripetal force model, the result of the gravity vector observation can be ameliorated, and the accuracy of attitude estimation enhanced under GPS-denied conditions can be maintained for a short time. Unfortunately, a large attitude error will be caused in maneuvering environments since the influence of linear acceleration was not considered in the gravitational acceleration estimation. Moreover, it is only used as attitude estimation without calculating the reliability of the velocity and position estimation. Li, X. et al. [20] proposed a method to estimate the external acceleration with the purpose of improving navigation performance under dynamic conditions. Marantos et al. [21] fully combined the visual algorithm and multisensor speed/position estimation with an adaptive complementary filter, which gave the algorithm a low computational complexity.

Compared with the convenience of rotorcraft to deploy intelligent algorithms related to vision, and lidar for simultaneous localization and mapping (SLAM) due to its low speed and more stable flight performance, there has been less work on the navigation and position of low-cost fixed-wing UAVs in the GNSS-denied environments because of the reasons mentioned above. Most of the previous works simply provide stable attitude output for UAVs in denied environments. The main contribution of this paper is to explore the provision of UAV stable state estimation in denied environments. The main work and innovations are as follows: (1) Based on [19], the filtering model further improves the accuracy of dynamic modeling, and an estimation algorithm of UAV nongravitational acceleration using airspeed and inertial sensors is proposed. We then further establish the mapping relationship between vehicle nongravitational acceleration and the vehicle attitude misalignment angle by combining the magnetometer. (2) Subsequently, the data of the barometer are fused to realize the stable tracking of the UAV in the altitude and lifting rate channels. (3) Aiming at the defect that the horizontal velocity and position errors of UAVs are easy to accumulate, a complementary filter for inertial navigation speed correction using airspeed assistance is designed, which greatly elevates the accuracy of the velocity position estimation of the vehicle.

**Figure 1:**
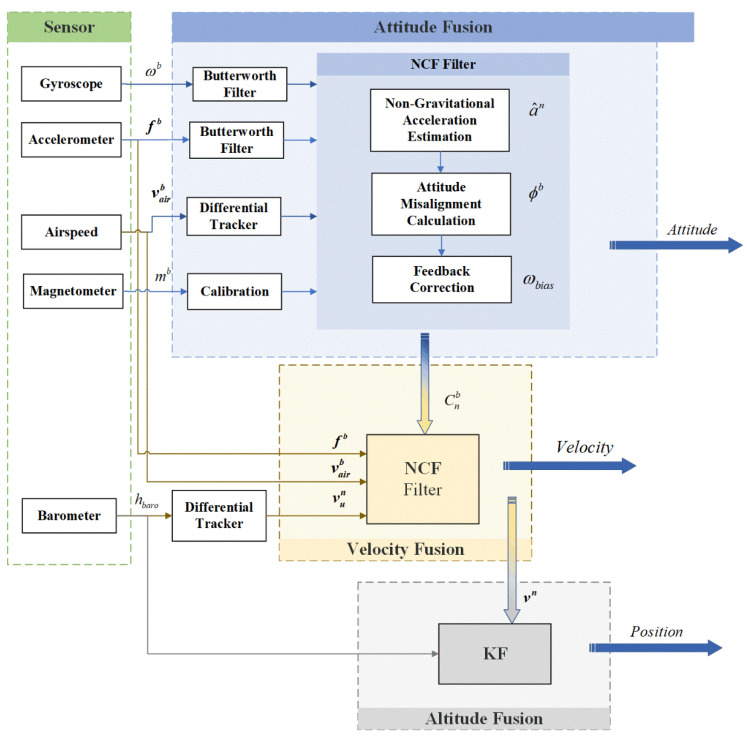
The framework of the multisensor fusing algorithm in this paper is divided into four parts. The green area shows the sensors we used in this study; the original sensor’s data will first be preprocessed, including using the Butterworth low-pass filter to reduce the high-frequency noise of the gyroscope and accelerometer, and using a differential tracker (DT) for the airspeed and barometer. The blue part is the attitude fusion frame, mainly divided into three parts: the main filter, the gravitational acceleration estimation described in Section 2.1, and the attitude misalignment angle calculation and the error feedback compensation are described in Section 2.2. Meanwhile, the yellow area presents the velocity fusion frame, and in Section 2.3, the filter which combines the barometer and airspeed is mainly described. The gray area represents the altitude fusion frame by the Kalman filter, which is established in Section 2.4.

The framework of the algorithm is shown as Figure 1. As a fully autonomous navigation solution, the algorithm proposed in this paper has been verified by real flight, which can be used as a key switch to airspeed compensation when GNSS is denied, and thus provides a more stable navigation result.

## 2. Airspeed-Aided Navigation Filter

### 2.1. Estimation of Nongravitational Acceleration

Euston, M. et al. [19] proposed a model that use airspeed and gyroscope measurements to estimate the centripetal acceleration of a vehicle, which the following equation can express.

(1) $${\hat{a}}_{n}=\omega_{ib}^{b}\times {\hat{V}}_{TAS}^{b},$$

where $\omega_{ib}^{b}$ is the 3-axis angular rate vector measured by the gyroscopes, ${\hat{V}}_{TAS}^{b}$ is the projection of the airspeed vector in the body frame, and ${\hat{a}}_{n}$ is the vector of centripetal acceleration.

Only considering centripetal acceleration in flight is not sufficient to describe the maneuvering process of the vehicle accurately. When the vehicle speeds up or slows down, the effect of linear acceleration also needs to be taken into consideration.

(2) $${\hat{a}}_{L}=\frac{d{\hat{V}}_{TAS}^{b}}{dt},$$

where ${\hat{a}}_{L}$ is the linear acceleration vector of the vehicle. The airspeed measured directly by the pitot tube is a scalar quantity defined in the velocity coordinate frame a. The longitudinal plane difference between frame a and the body frame b is the angle of attack α, and the horizontal difference is the sideslip angle β.

Accurate angle-of-attack calculation requires unique sensors. The angle of attack can be estimated by flight dynamics approximation on low-cost UAVs. We just consider the vertical channel of the UAV, which can be described as follows:

(3) $$\alpha =\varphi -\theta \;\theta \;=\arcsin\left(\frac{v_{h}}{v}\right),$$

where θ is the flight path angle, which can be calculated from the triaxial velocity, and φ is the pitch Angle. The airspeed vector can be described in the airflow frame as $v_{air}^{a}={\left[\begin{array}{ccc} 0 & v_{air} & 0 \end{array}\right]}^{T}$, the transfer to body frame as $v_{air}^{b}=C_{a}^{b}v_{air}^{a}$, where $C_{a}^{b}$ denotes the attitude rotation matrix from the airflow frame to the body frame. Therefore, $v_{air}^{b}={\left[\begin{array}{ccc} -vcos\alpha cos\beta & vcos\alpha cos\beta & -vsin\alpha \end{array}\right]}^{T}$

$$C_{a}^{b}=\left[\begin{array}{ccc} cos\beta & -cos\alpha cos\beta & -sin\alpha sin\beta \\ sin\beta & cos\alpha cos\beta & sin\alpha cos\beta \\ 0 & -sin\alpha & cos\alpha \end{array}\right]$$

For small fixed-wing UAVs, the angle of attack and the sideslip angle are difficult to measure directly by sensors because accurate measurements require atmospheric parameter sensors, but they are not suitable for small vehicles. We notice that if the sideslip angle β in flight is approximately no more than 10 degrees (in reference [22], as for small fixed-wing UAVs, the sideslip angle estimation is no more than 5 degrees), and the cosine of 10 degrees is equal to 0.9848, it makes only 1.52% velocity errors if we assume the effect of the sideslip angle is ignored. In contrast, the angle of attack α is the angle between the incoming direction of the flow vector and chord line of an airfoil. As the angle of attack increases, the relative lift of the airfoil increases. When the UAV makes a turn, additional centripetal acceleration is provided by increasing the angle of attack. To ensure centripetal acceleration at the turn, the vehicle enters a Bank-to-Turn (BTT) inclined turn mode where the increased lift from the wing is decomposes into a vertical component and a horizontal component. In order for the vehicle to maintain altitude, the vertical component of lift must counteract gravity, which requires increasing α to gain additional lift. So, the angle of attack α cannot be ignored, especially when the vehicle in turning.

**Figure 2:**
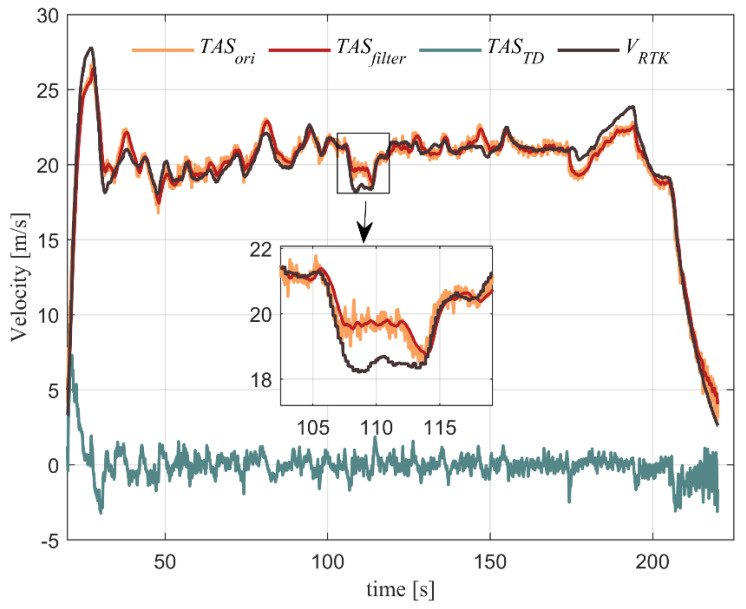
The comparisons between the true airspeed (TAS) measured by pilot tube and the ground velocity measured by RTK. The orange line shows the original signal of TAS. The red line and the green line represent the TAS data of the filtered and differential tracker (TD) modules, respectively.

Obviously, the field winds are dynamic and inevitable. Due to the fact that small fixed-wing UAVs are lightweight, they are not suitable to fly in high field wind. Moreover, the wind speed is as hard to estimate as the angle of attack or sideslip, so we try to ignore the effect of the wind. Figure 2 shows the comparison between the true airspeed and the ground velocity measured by RTK in real flight.

The linear acceleration of the vehicle can be calculated from the differentiation of the linear velocity. Random noise inevitably exists in the pitot airspeed measurement, which leads to an additional error in acceleration estimation. The function of the differential tracker (TD) of the Active Disturbance Rejection Control (ADRC) is to extract differential signals properly from those polluted by noise, so the second-order differential tracker [23] in the ADRC is used to achieve data filtering and differential signal extraction.

The second-order differential tracker is described as follows:

(4) $$\{\begin{array}{c} x_{1}\left(k+1\right)=x_{1}\left(k\right)+Tx_{2}\left(k\right)\\ x_{2}\left(k+1\right)=x_{2}\left(k\right)+T\cdot fst \end{array},$$

where $x_{1}\left(k\right)$ tracks the original signal, $x_{2}\left(k\right)$ calculates the differential value of the original signal, and the fst is calculated as follows:

(5) $$\{\begin{array}{l} \delta =rh\\ \delta_{0}=\delta h\\ y=x_{0}-u+hx_{2}\\ a_{0}=\sqrt{\delta^{2}+8r\left|y\right|}\\ a=\{\begin{array}{l} x_{2}+\frac{y}{h},\left|y\right|\leq \delta_{0}\\ x_{2}+0.5\left(a_{0}-\delta \right)sgn\left(y\right),\left|y\right|>\delta_{0} \end{array}\\ fst=\{\begin{array}{l} -r\frac{a}{\delta },\left|a\right|\leq \delta \\ -rsgn\left(a\right),\left|a\right|>\delta \end{array} \end{array},$$

here, T is the period of the input signal and h is the filter factor; when h=T, the algorithm is close to the first-order difference. The higher the value of h, the better the filter effect will be. Still, it will bring the corresponding time delay. Factor r is the rate factor, which can be used to adjust the tracking speed. The speed will raise as the factor r increases, but the signal noise will be amplified.

Based on the above equation, the vehicle acceleration model is:

(6) $$\hat{a}=\omega_{ib}^{b}\times {\hat{V}}_{TAS}^{b}+\frac{dv_{TAS}^{a}}{dt}$$

### 2.2. Attitude Calculation Model Based on External Acceleration Correction

The inertial navigation-specific force equation under local horizontal frame is written as:

(7) $${\dot{v}}^{n}=C_{b}^{n}f^{b}+g^{n}-\left(2\Omega_{ie}^{n}+\Omega_{en}^{n}\right)v^{n},$$

here, the corner marks b and n, respectively, denote the East-North-Up (ENU) and the body frame. $V^{n}$ represents the speed of the vehicle, $f^{b}$ represents the specific force vector under the body frame, $g^{n}$ denotes the gravity field vector in the ENU frame, and $C_{b}^{n}$ is the coordinate transformation matrix from the body frame to the ENU frame. Moreover, $\Omega_{ie}^{n}$ denotes the Earth-rotation skew-symmetric matrix in the local navigation frame, and $\Omega_{en}^{n}$ denotes the transport rate skew-symmetric matrix from the rotation of the local-frame to the center frame, which can be neglected due to the short flight span.

(8) $$a^{n}=C_{b}^{n}f^{b}+g^{n},$$

here, $a^{n}$ is the nongravitational acceleration of the vehicle in the local horizontal frame. The $C_{b}^{n}$ is the theoretical value of the attitude rotation matrix. Due to the measurement error of the gyroscope, the relationship between $C_{b}^{n}$ and the real calculated value ${\tilde{C}}_{b}^{n}$ is shown as follows [24]:

(9) $$a^{n}=\left(I+\phi^{n}\times \right){\tilde{C}}_{b}^{n}f^{b}+g^{n},$$

here, $\phi^{n}$ is the projection of the attitude misalignment angle vector in the ENU frame.

Because the gyroscope bias accumulates large attitude errors over time, it is necessary to estimate and compensate the gyroscope bias in real-time. The bias of the accelerometer is usually small, and we only want to calculate the nongravitational acceleration of the vehicle, which is not cumulative, so we assume that the accelerometer measurement error is negligible as $f^{b}\approx {\tilde{f}}^{b}$. Then, the equation written as:

(10) $${\tilde{f}}^{n}\times \phi^{n}=g^{n}+{\tilde{f}}^{n}-a^{n}$$

The left ${\tilde{f}}^{n}$ can be approximated as ${\tilde{f}}^{n}\approx a^{n}-g^{n}$, where $g^{n}={\left[\begin{array}{ccc} 0 & 0 & -g \end{array}\right]}^{T}$.

Construct the above equation in component form as:

(11) $$\left[\begin{array}{c} a_{E}\\ a_{N}\\ a_{U}-g \end{array}\right]\times \left[\begin{array}{c} \phi_{E}\\ \phi_{N}\\ \phi_{U} \end{array}\right]=\left[\begin{array}{c} f_{E}-a_{E}\\ f_{N}-a_{N}\\ f_{U}-g-a_{U} \end{array}\right]$$

Due to rank((a−g)×)=2<3, Equation (11) can only solve two attitude misalignment angles. Because horizontal acceleration only provides information about horizontal misalignment angle, the heading misalignment angle cannot be observed. Accordingly, make $\phi_{U}=0$.

Then, the misalignment angle under the navigation system can be described as Equation (12). The $e_{3}={\left[\begin{array}{ccc} 0 & 0 & 1 \end{array}\right]}^{T}$ presents the z-axis unit vector. Convert to the body frame:

(12) $$\phi^{b}=\frac{f^{b}-a^{b}}{a_{U}-g}\times \left(C_{n}^{b}e_{3}\right)\;=\frac{f^{b}-a^{b}}{a_{U}-g}\times C_{3}^{T},$$

where $C_{3}$ is the third-row vector of the $C_{b}^{n}$. The specific force $f^{b}$, the acceleration of air velocity measurement $a^{b}$, and the acceleration of gravity g are all vectors whose errors do not diverge with time. Since the attitude rotation matrix $C_{n}^{b}$ is obtained by integrating the angular rate, it will generate cumulative errors over time. The cumulative errors can be converted into the projection of the horizontal attitude misalignment angle $\phi_{\varphi \gamma }^{b}$ under the body frame by multiplying these two vectors.

For the yaw channel, the magnetometer complementary filter is adopted.

(13) $$\phi_{\psi }^{b}=\frac{m^{b}}{\left|m^{b}\right|}\times \left(C_{n}^{b}{\hat{m}}^{n}\right),$$

(14) $$\phi^{b}=\phi_{\varphi \gamma }^{b}{\left[\begin{array}{ccc} 1 & 1 & 0 \end{array}\right]}^{T}+\phi_{\psi }^{b}{\left[\begin{array}{ccc} 0 & 0 & 1 \end{array}\right]}^{T},$$

similarly, $\phi_{\psi }^{b}$ is the projection of the heading error angle under the system, and $m^{b}$ is the triaxial magnetometer measurement vector.

Take the misalignment angle into the complementary filter, and the measurement error generated by the gyroscope can be corrected in the next step. The negative feedback model of the complementary filters uses a PI controller. It can be defined as:

(15) $$\omega_{bias}=k_{p}\phi_{b}+k_{I}\int \phi_{b}.$$

the gains $k_{p}$ and $k_{I}$ are proportional and integral gains, respectively. Adjusting the appropriate $k_{p}$ gain can make the system track the angular motion quickly and compensate the attitude misalignment angle continuously.

(16) $$q_{k+1}=q_{k}⊗\Delta q_{k},$$

here, $\Delta q_{k}$ is the delta quaternion, which can be defined as:

(17) $$\Delta q_{k}=cos\frac{\Delta \theta }{2}+\frac{\Delta \theta }{\left\|\Delta \theta \right\|}sin\left(\frac{\Delta \theta }{2}\right),$$

(18) $$\Delta \theta =\left(\omega_{ib}^{b}+\omega_{bias}^{b}\right)\cdot \Delta T$$

At high sampling times, the delta angle Δθ from k moment to k+1 moment is usually tiny and can be approximated as follows:

(19) $$q_{k}\approx \;{\left[\begin{array}{cccc} 1 & \frac{\Delta \theta_{x}}{2} & \frac{\Delta \theta_{y}}{2} & \frac{\Delta \theta_{z}}{2} \end{array}\right]}^{T}$$

The three-axis attitude can be solved from the quaternion.

### 2.3. Adaptive Complementary Fusion in Horizontal Channel

UAV velocity estimation can be obtained recursively through a specific force equation:

(20) $$v^{n}=\int \left(C_{b}^{n}f^{b}+g^{n}\right)dt$$

Due to the errors of inertial measurement and attitude calculation accumulation with time, the velocity position estimation will become meaningless over a long time. A complementary filter is used to smooth and correct the horizontal velocity by airspeed measurement.

(21) $${\hat{v}}_{E}=v_{TAS}sin\left(\psi \right)+{\tilde{v}}_{windE}\;\;{\hat{v}}_{N}=v_{TAS}cos\left(\psi \right)+{\tilde{v}}_{windN}$$

Using the above Equation (21), the airspeed can be converted to the local frame.

(22) $$v_{I}^{k,N/E}=v_{I}^{k^{-},N/E}+K_{TAS}^{v}\left({\hat{v}}_{TAS}^{k^{-},N/E}-v_{I}^{k^{-},N/E}\right),$$

here, $K_{TAS}^{v}$ is the gain of the complementary filter fused with airspeed. The state estimator parameters have adaptive functions to obtain the best performance based on sensor characteristics.

We use the following function to adjust the gain $K_{TAS}^{v}$ and use an activation function to smooth the gain $K_{TAS}^{v}$ switching process of the observer. The adaptive strategy is given in the following equation.

(23) $$K_{TAS}^{v}=\{\begin{array}{c} 0,\;if\;t\leq t_{0}\\ \frac{G}{1+e^{-\left(t-t_{0}-t_{1}\right)}},if\;t>t_{0} \end{array},$$

where $t_{0}$ is the time switching threshold, and the velocity solved by the inertial navigation algorithm can maintain a low error when $t\leq t_{0}$. This error is lower than that in the direct estimation of the ground velocity from the airspeed, so the gain should be set to a low value, indicating complete trust in the vehicle velocity calculated by the inertial integration. When $t>t_{0}$, the inertial velocity integration error gradually accumulates and is greater than the estimated value using the airspeed, and at this time should improve the gain correction effect. G is the gain value and $t_{1}$ factors the control the curve smoothness.

### 2.4. High Channel Kalman Filtering Model

For the flight control level, the fixed-wing UAVs require a high accuracy of altitude position and lift rate, which affects the climbing, landing, and cruising performance of the UAV. The GNSS-denied environments are limited by the inertial navigation accuracy and the inability to measure the local gravitational acceleration precisely. Using low-precision inertial guidance alone for altitude solving, the altitude error will diverge significantly over time. The barometer is susceptible to high-frequency noise from the atmospheric environment, so fusing the barometer to the inertial navigation for correction is necessary.

The inertial sensor, ADIS16488B, has a built-in barometer to measure static atmospheric pressure. The simplified conversion equation of atmospheric pressure to altitude is shown below [25]. The performance parameters of the barometer are shown as Table 1.

(24) $$H_{b}=44,300\times \left[1-{\left(\frac{P_{s}}{P_{0}}\right)}^{\frac{1}{5.255}}\right],$$

here, $H_{b}$ is the altitude we require. $P_{0}=1.01325\;\mathrm{bar}\;$ is the value of standard atmosphere and $P_{s}$ is the measured value of the barometer.

Barometer measurement error is mainly affected by airflow intensity and temperature, and the changes of temperature make the barometric output drift. Using the temperature control system in the flight control can achieve the heat balance before the data fusion solution. In contrast, the ADIS16488B sensor embedded in the flight control component has been indirectly isolated from airflow, so the error correlation is significantly reduced. The height measurement error and the rate of change error correlation use the first-order Markov (Markov) process.

(25) $$\delta {\dot{h}}_{baro}=-\frac{1}{\tau_{baro}}\delta h_{baro}+\omega_{baro}\;\delta {\dot{v}}_{h_{baro}}=-\frac{1}{\tau_{h_{baro}}}\delta v_{h_{baro}}+\omega_{h_{baro}},$$

where $\delta h_{baro}$ and $\delta v_{h_{baro}}$, respectively, denote the error of barometer height and lift rate. $\tau_{baro}$ and $\tau_{h_{baro}}$ denote the correlation time coefficient. $\omega_{baro}$ and $\omega_{h_{baro}}$ represent white noise.

(26) $$\begin{array}{c} \delta {\dot{v}}^{n}=f_{sf}^{n}\times f+v^{n}\times \left(2\delta \omega_{ie}^{n}+\delta \omega_{en}^{n}\right)-\left(2\omega_{ie}^{n}+\omega_{en}^{n}\right)\times \delta v^{n}\\ +\delta f_{sf}^{n}+\delta g^{n}\\ \approx f_{sf}^{n}\times f+\delta f_{sf}^{n}+\delta g^{n} \end{array}$$

The above Equation (26) is the error transfer model of the inertial navigation in the altitude channel. After ignoring the small error caused by rotation, the error equation of inertial navigation in the altitude channel is established as Equation (27).

(27) $$\delta {\dot{v}}_{U}=-f_{N}\phi_{E}+f_{E}\phi_{N}+\delta g+\Delta_{U},$$

here, $\phi_{N}\;and\;\phi_{E}\;$ are the horizontal misalignment angle and δg denotes the gravity acceleration error. $\Delta_{U}$ represents the altitude channel bias of the accelerometer. For the horizontal misalignment angle, we consider that it has been compensated in Equation (15). In addition, the error of the gravitational acceleration term is also ignored, so we consider that the velocity error in the altitude channel comes from the bias of the accelerometer. We describe the z axis velocity error by using the first-order Markov process.

(28) $$\begin{array}{c} \delta {\dot{v}}_{U}=-\frac{1}{\tau_{\Delta }}\delta v_{U}+\omega_{U}\\ \delta {\dot{h}}_{INS}=\delta v_{U}+\omega_{h} \end{array},$$

where $\delta v_{U}$ and $\delta h_{INS}$ denote the inertial navigation z-axis velocity error and the altitude error, respectively. $\tau_{\Delta }$ is the correlation time coefficient. $\omega_{U}$ and $\omega_{h}$ are the white noise.

According to Equations (25) and (28), the state equation of the system is established as follows:

(29) $$\dot{X}\left(t\right)=F\left(t\right)X\left(t\right)+W\left(t\right)$$

The state is $X\left(t\right)={\left[\begin{array}{cccc} \delta v_{U} & \delta v_{baro} & \delta H_{INS} & \delta H_{baro} \end{array}\right]}^{T}$

The system translation matrix is:

$$F\left(t\right)={\left[\begin{array}{cccc} -\frac{1}{\tau_{\Delta_{z}}} & 0 & 0 & 0\\ 0 & -\frac{1}{\tau_{v_{baro}}} & 0 & 0\\ 1 & 0 & 0 & 0\\ 0 & 0 & 0 & -\frac{1}{\tau_{h_{baro}}} \end{array}\right]}_{4\times 4}$$

The system observation equation is:

Z(t)=H(t)X(t)+V(t),

here,$\;H\left(t\right)=\left[\begin{array}{cccc} 1 & -1 & 0 & 0\\ 0 & 0 & 1 & -1 \end{array}\right]$.

**Figure 3:**
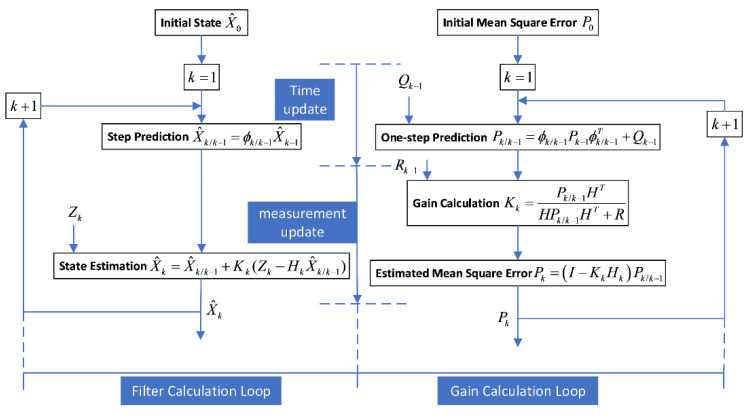
The flow chart of the Kalman filter. At each calculated step size, the Kalman algorithm runs in two steps: time updating and measurement updating. The time-update step predicts the navigation states vector and its covariance matrix by propagation through a model of the system dynamics. The measurement-update uses the data from the sensors and is incorporated to correct the prediction and output an optimal estimation by calculating the optimal Kalman gain. The system error state can be estimated by the Kalman filter, then the system error can be corrected by closed-loop feedback.

The system equation is discretized and solved by the Kalman filter equation. The algorithm flow chart of the filter is shown as Figure 3:

(30) $${\hat{v}}_{U}=v_{U}-K_{1}\delta v_{U}\;{\hat{H}}_{INS}=H_{INS}-K_{2}\delta H_{INS},$$

where $K={\left[\begin{array}{cc} K_{1} & K_{2} \end{array}\right]}^{T}$ is the error feedback coefficient. Adjusting the appropriate gain can make the signal smoother.

## 3. Experiment

**Figure 4:**
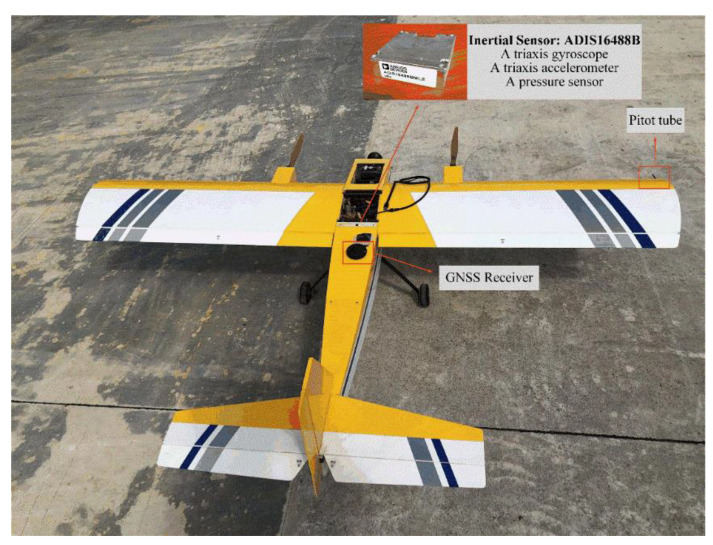
The experimental platform of the small fixed-wing UAV.

The small fixed-wing drone was used for experiments to verify the correct functionality in a practical scenario, and the vehicle is shown in Figure 4 and the UAV parameters are shown in Table 2. As an algorithm-verified vehicle, the UAV flight is fully autonomous on route. The flight control system was synthesized on an OMAP-L138 C6000 using the MATLAB/Simulink code generation design tool to build the embedded code. A serial-to-parallel interface (SPI) was developed to connect directly with the ADIS16488B sensor, which consisted of the three-axis gyro, three-axis accelerometer, three-axis magnetometer, and the barometer sensor. This IMU error indicator is shown in Table 3. We can see in Figure 2 that the maximum wind speed under this experiment is 3 m/s.

**Figure 5:**
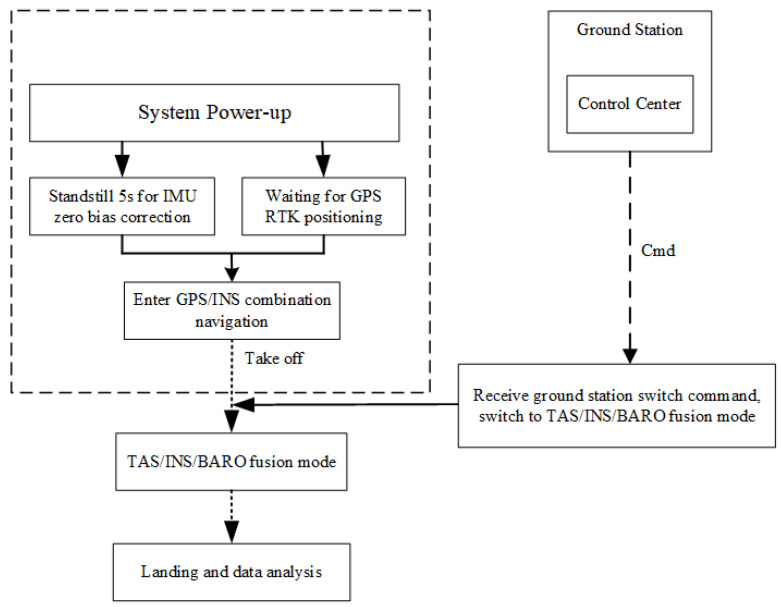
The system experimental task flow chart. The flight control system needs to wait for 5 s for bias correction and self-alignment after power-on. When receiving RTK signal, the system enters GNSS/INS cooperative mode. The algorithm switches to TAS/INS/BARO fusion mode during the flight after receiving the navigation switching command from the ground station.

Both the flight control algorithm and the multisensor fusion algorithm are arranged into the flight control hardware platform. The flow chart of the experimental tasks of the whole system is shown in Figure 5.

The main parameter values of the algorithm are shown in Table 4. Since no higher accuracy inertial navigation is applied as the flight attitude reference, the combined GNSS/INS mode is still used for comparison and analysis in the UAV navigation control loop. The algorithm result data of the fused TAS/INS/BARO are saved to the flight log, and the vehicle completes the maneuvers of turning, circling, pulling up, and descending in the air independently to verify the navigation accuracy. Finally, the flight log is read after the vehicle lands for data comparison and analysis. The algorithm proposed in this paper is compared with the two-vector EKF model (denoted as EKF/TAS) proposed by [16], the centripetal force compensation model fused with airspeed presented in [19] (denoted as NCF/TAS), and the ACF model with adaptive adjustment weights estimated from external acceleration (denoted as ACF). Since there is little difference between the offline solution and online real-time calculation, we use offline processing to compare the accuracy of different algorithms. The results of the GPS/INS combination are used as true values for error calculation analysis. The results are shown below.

**Figure 6:**
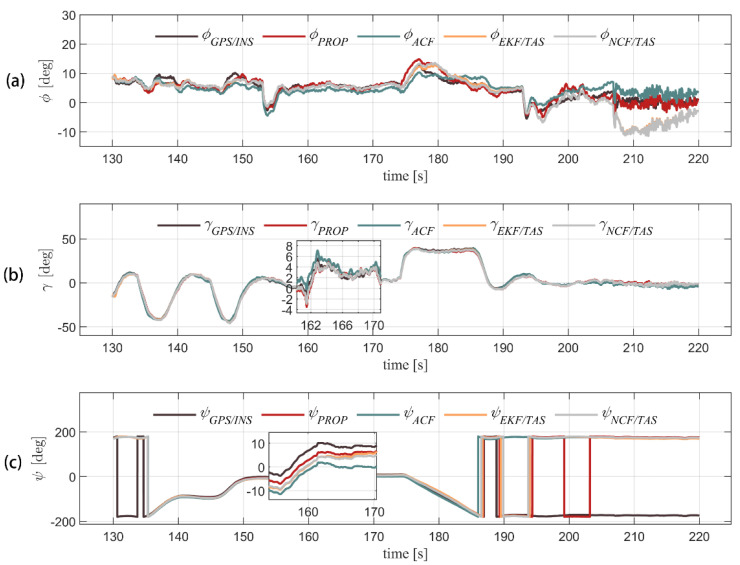
Three-axis attitude solution of five algorithms in flight is shown for pitch, roll, and yaw angle (from (**a**–**c**), respectively).

**Figure 7:**
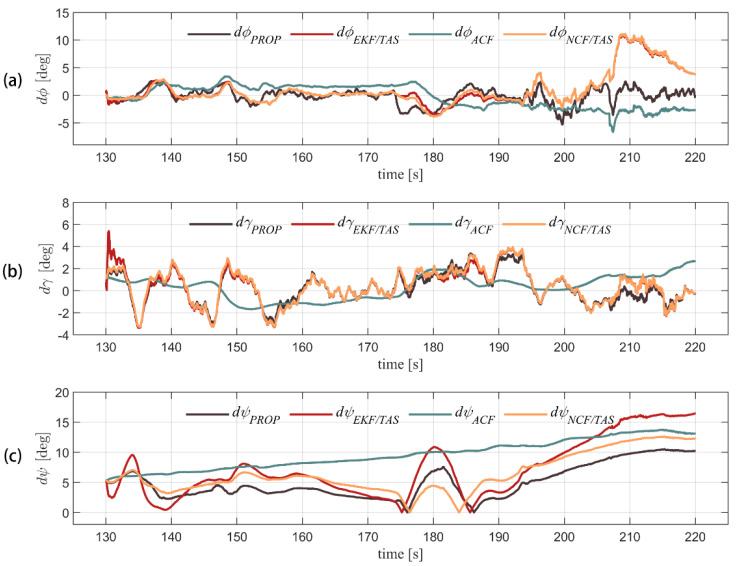
GPS/INS integrated navigation result is used as true values. Three-axis attitude error of other four algorithms in flight is shown for pitch, roll, and yaw error angle (from (**a**–**c**), respectively).

Figure 6 and Figure 7, respectively, show the Euler angles calculation and attitude error comparison of the four algorithms. It can be seen from Table 5 and Figure 7 that the attitude error of the ACF is relatively stable, but it is challenging to correct the attitude error directly with the accelerometer because the external acceleration estimation of the air velocity is not accurate. It is obvious that attitude errors accumulate over time. EKF/TAS, NCF/TAS, and the algorithm proposed in this paper add airspeed measurement into the filter. Due to the influence of wind, higher fluctuations in attitude error can be seen. As EKF/TAS and NCF/TAS algorithms do not consider the effect of linear acceleration, the difference in attitude error among the three is not significant when the UAV flight speed is close to constant. At about 200 s, the UAV began to descend. It can be seen that the pitch error increased rapidly, and the instantaneous maximum reached nearly 11°. In 190 s, it can be seen in Table 5 that the MAE and RMSE of the roll error are 1.1388° and 1.4195°, the MAE and RMSE of pitch error are 1.1114° and 1.4672°, and the MAE and RMSE of yaw error are 4.7935° and 5.5443°, respectively. The yaw angle preformed worse due to the accuracy of the yaw estimation and was affected by the precision of the magnetometer. (It is difficult to calibrate the magnetic field around the UAV accurately, and the magnetometer is highly susceptible to disturbances from the electromagnetic environment, which makes the heading angle accuracy worse.) Generally, the error of the proposed algorithm is stable. The results suggest that this algorithm can adapt to the attitude estimation of the UAV under flight dynamics.

**Figure 8:**
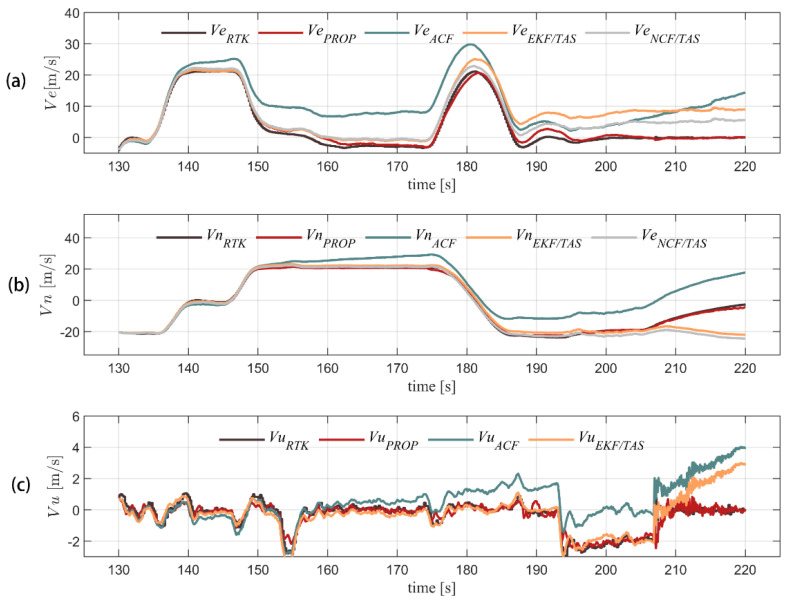
Three-axis velocity solution of five algorithms in flight is shown for east, north, and up velocity (from (**a**–**c**), respectively). In order to find difference of horizontal position, NCF/TAS and what we proposed use the same height model, so the curve of NCF/TAS is not drawn in the bottom figure.

**Figure 9:**
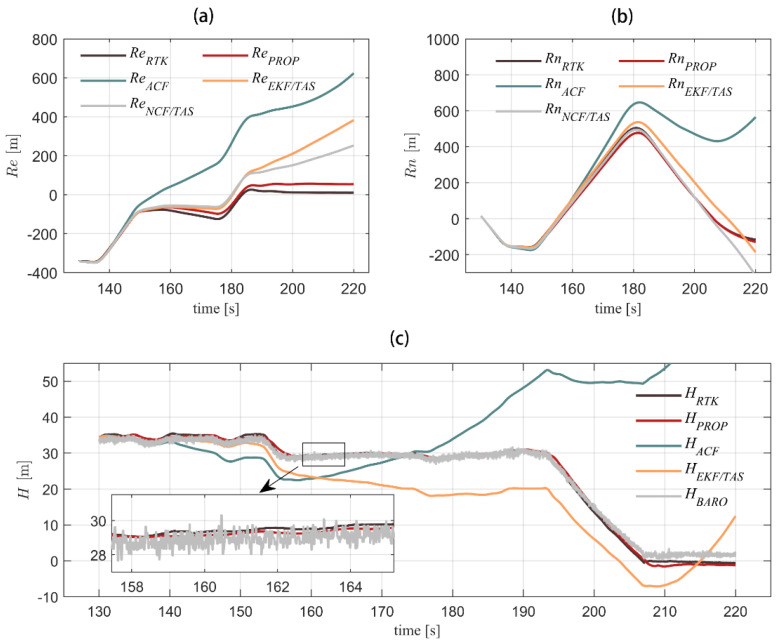
Three-axis position solution of five algorithms in flight is shown for east position, north position, and altitude (from (**a**–**c**), respectively). In order to find difference of horizontal position, NCF/TAS and what we proposed use the same height model, so the curve of NCF/TAS is not drawn in the figure (**c**).

**Figure 10:**
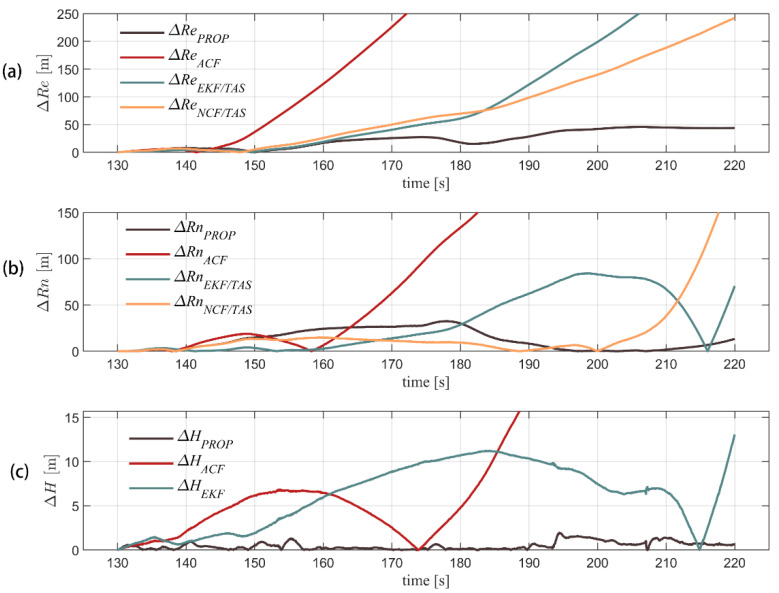
GPS navigation result is used as true values. Three-axis position error of the other four algorithms in flight is shown for east position, north position, and altitude error angle (from (**a**–**c**), respectively).

**Figure 11:**
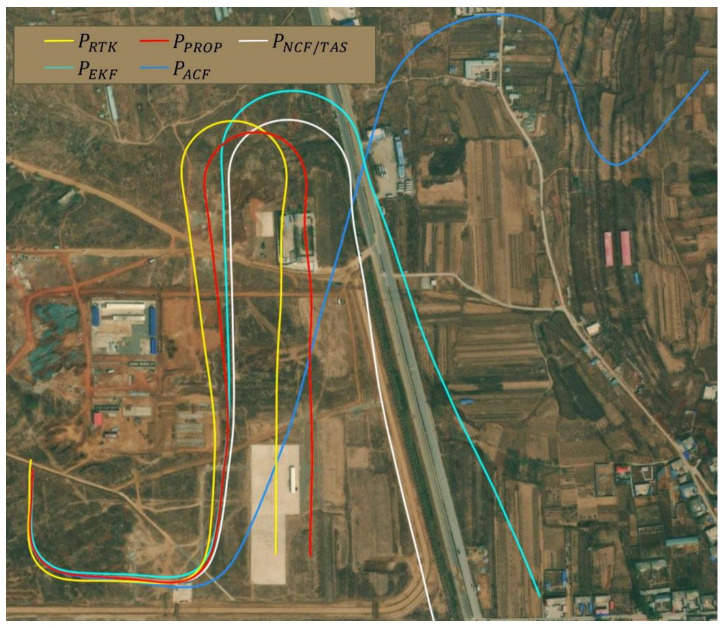
Comparison of 190 s long trajectories obtained from different algorithms, and what we proposed is the most accurate tracking.

Figure 8 shows the comparison among the velocity solutions of the different algorithms and RTK truth values. The horizontal velocity of the model in this paper can well track RTK velocity measurement with an error within 2 m/s. The defect of horizontal velocity error accumulated over time can be changed by using complementary filter and integrated airspeed. The vertical channel is integrated with a barometer to gain the maximum error of 0.5 m/s, which has obvious advantages over the other three algorithms. Figure 9 shows a comparison of the positions settled by RTK and the other four algorithms. Figure 10 compares the position errors of the different algorithms, while Figure 11 depicts the comparison between the track solution and the real track. It can be seen that the new algorithm can better track the position of the RTK. The error of 30 s (about 600 m flying) in the horizontal channel flight is within 30 m, the error of 90 s (about 1800 m flying) is within 50 m, and the average error of the height channel is 0.5 m, with higher accuracy than the other three. We also notice that during 140–190 s, the north position error of the proposed algorithm is a little more than the ACF and NCF/TAS. The low-precision INS position error transfer equation is established as Equation (31).

(31) $$\Delta {\dot{R}}_{n}=\int (f_{U}\phi_{E}-f_{E}\phi_{U}-2\omega_{U}{\Delta v}_{E})dt,$$

where the $\phi_{E}$ and $\phi_{U}$ is the east and up direction attitude misalignment angle, respectively, which are the main source of north position errors. From Figure 7 and Figure 11, after the first turn, the roll angle error caused by random wind disturbance is converted to the north position cumulative error. After 160 s, the adaptive complementary filter in Equation (22) makes sense, and the north error stops growing. The algorithm eliminates the accumulated error caused by attitude misalignment error to position as much as possible.

We also compared the offline computing efficiency of the four algorithms. The host computer with the 3.2 GHz AMD Ryzen 7 5800H CPU was used to run the four models with MATLAB 2021A. The total running time was set to 200 s with each step length of 0.005 s. Table 6 shows the comparison of the running time and its actual ratio among the different algorithms.

As can be seen from the Table 6, the adaptive complementary filter algorithm (ACF) has the highest computational efficiency, accounting for only 2.12% of the actual operating time of the algorithm. It is usually a nice choice for a lightweight sensor fusion algorithm. The value of the fusion airspeed nonlinear complementary filter (NCF/TAS) is 3.21%. Due to differential tracking of airspeed data and other algorithm modules, the ratio of the running time in the proposed algorithm is 4.09%, slightly higher than ACF and NCF/TAS. Although the computation time is slightly longer, this processing improves the navigation accuracy, and this algorithm can be deployed in our flight control equipment for real autonomous flight verification. As for the EKF algorithm, it requires several high-dimensional matrix operations and is not superior in operational efficiency, accounting for 6.31%. The proposed algorithm consumes fewer computing resources than the EKF/TAS and can provide the higher precision attitude, position, and speed solutions than the EKF/TAS algorithm. The performance of the fusion algorithm is very satisfactory.

## 4. Conclusions

This paper proposes a robust and universal sensor fusion algorithm, including an IMU, barometer, magnetometer, and airspeed sensor. The contributions of this paper include the following: (1) We use airspeed to improve the estimation accuracy of the nongravitational acceleration of vehicle, subsequently, to optimize the nonlinear complementary filter model of the vehicle’s attitude misalignment angle based on observability derivation, which can adapt to the state estimation accuracy of the UAV under different maneuvers. In the flight test, the MAE and RMSE of roll error are 1.1388° and 1.4195°, the MAE and RMSE of pitch error are 1.1114° and 1.4672°, and the MAE and RMSE of yaw error are 4.7935° and 5.5443°, respectively, and, when compared with the more commonly used EKF algorithm, is improved. (2) At the level of the horizontal velocity fusion, a complementary filtering model using airspeed correction is established to suppress the accumulated errors caused by the calculation speed of INS. (3) At the height level, the Kalman filter model is designed using barometer data so that the vehicle can obtain the accurate solution of the lift rate and altitude without GNSS. The average error of the height channel is 0.5 m, and the maximum error of the lift rate is 0.5 m/s. This design idea uses a cascade fusion strategy that combines the benefits of an individual systems model using a cascade fusion strategy, combining the advantages of a single system. Compared against the other three conventional methods, the proposed method shows superior performance, providing good attitude velocity and position estimation, even in GNSS-denied environments. In addition, the algorithm proposed in this paper consumes lower computing resources and is suitable for common embedded systems. Taken as a whole, the new approach provides a feasible solution for the navigation and positioning of small UAVs, as much as possible in GNSS-denied environments; however, the result is still not very precise. In further works, we will continue to explore multiple fusion navigation technologies, and explore the use of low-cost camera sensors to enhance the robustness and fault tolerance of navigation systems.

## Figures and Tables

**Table 1:** The performance parameters of the barometer we use. The errors of the barometer mainly consist of the measurement error and measurement noise. The absolute error of measurement reaches 40 m.

Measurement Error Parameters	The Parameter Values	
Measurement range	300−1100 mbar	−700∼9165 m
Measurement error	4.5 mbar	About 40 m (About 500 m above sea level)
Measurement noise	0.025 mbar	About 0.2 m (About 500 m above sea level)

**Table 2:** Parameters of fixed-wing UAV.

Parameters	Value
Total Weight	6.9 kg
Span	2100 mm
Body length	1620 mm
Power	Electric drive

**Table 3:** Specifications of ADIS16488B.

Parameters	Typical Value
In-Run Bias Stability of Gyroscope	6.25°/hr
Angular Random Walk	$0.3{}^{\circ}/\sqrt{\mathrm{hr}}$
In-Run Bias Stability of Accelerometer	0.1 mg
Velocity Random Walk	$0.029\;m/s/\sqrt{\mathrm{hr}}$

**Table 4:** The main parameter values of the algorithm we used in the following experiment.

Categories	Variable	Definition	Value
Differential tracker filter	T	Period of the input signal	0.005
	h	Filter factor	0.15
	r	Rate factor	900
Attitude calculation model	$k_{p}$	The ϕ γ compensation gain	0.05
	$k_{I}$	The ϕ γ compensation integral gain	0.01
	$k_{p}$	The ψ compensation gain	0.2
	$k_{I}$	The ψ compensation integral gain	0.01
Horizontal channel adaptive complementary fusion	$K_{TAS}^{v}$	The gain factor of the complementary filter	0.9
	G	The gain value	1
	$t_{0}$	The time switching threshold	30
	$t_{1}$	The curve smoothness control factor	10
High Channel Kalman Filtering	Q	The error covariance matrix	diag ([0.1, 0.5, 0.1, 2])^2^
	R	The measurement noise covariance matrix	diag ([1, 10])^2^
	$P_{0}$	The initial covariance matrix	diag ([0.1, 1, 0.1, 10])^2^
	$K_{1}$	The error feedback coefficient of lift rate	0.2
	$K_{1}$	The error feedback coefficient of altitude	0.8

**Table 5:** In the experiment, the attitude error evaluation indexes of MAE (Mean Absolute Error) and RMSE (Root Mean Square Error) calculated by the proposed fusion algorithm are compared with those calculated by the adaptive complementary filter (ACF), the two-vector extended Kalman filter fused with airspeed (EKF/TAS), and the nonlinear complementary filter fused with airspeed (NCF/TAS).

Methods		Attitude (deg)		
		Roll (γ)	Pitch (ϕ)	Yaw (ψ)
ACF	MAE	0.9871	1.8859	9.4879
	RMSE	1.1324	2.0623	9.8084
EKF/TAS	MAE	1.2389	1.8450	7.0168
	RMSE	1.5633	3.1493	8.4507
NCF/TAS	MAE	1.2564	1.8770	6.2642
	RMSE	1.5557	3.2114	7.0386
PROP	MAE	1.1388	1.1114	4.7935
	RMSE	1.4195	1.4672	5.5443

**Table 6:** Comparison of the computational efficiency among different algorithms.

Algorithm	Total Time (s)	The Ratio of the Actual Running Time of the Algorithm to the Total Simulation Time
ACF	4.2402	2.12%
EKF/TAS	12.6251	6.31%
NCF/TAS	6.4150	3.21%
PROP	8.1774	4.09%

## Data Availability

The data presented in this study are available on request from the corresponding author. The data are not publicly available due to confidentiality agreement for the project.
